# Posterior cervical full-endoscopic technique for the treatment of cervical spondylotic radiculopathy with foraminal bony stenosis: A retrospective study

**DOI:** 10.3389/fsurg.2022.1035758

**Published:** 2023-01-04

**Authors:** Meng Shi, Cong Wang, Huihao Wang, Xiaoqing Ding, Juntao Feng, Lin Zhou, Yuwei Cai, Zhongxiang Yu

**Affiliations:** ^1^ Department of Orthopaedics, ShuGuang Hospital, Shanghai University of Traditional Chinese Medicine, Shanghai, China; ^2^ Department of Radiology, ShuGuang Hospital, Shanghai University of Traditional Chinese Medicine, Shanghai, China

**Keywords:** posterior cervical full-endoscopic technique, cervical spondylotic radiculopathy, foraminal bony stenosis, effectiveness, cervical spine stability, retrospective study

## Abstract

**Objective:**

In the treatment of cervical spondylotic radiculopathy (CSR), spinal endoscopy has been developed vigorously in the past 30 years. However, its effectiveness and subsequent problem of cervical spine stability have always been the controversial hotspots. This study aims to conduct a retrospective study using posterior cervical full-endoscopic technique for the treatment of CSR with foraminal bony stenosis, and evaluate its clinical effect and application value.

**Methods:**

All 22 patients treated for CSR with foraminal bony stenosis using posterior cervical full-endoscopic technique were analyzed since Dec 1, 2016, to Apr 30, 2020. The data collection included operation time, length of stay, wound healing, surgical complications, visual analog scale (VAS), Japanese Orthopaedic Association (JOA) scores, intervertebral foramen diameter, intervertebral foramen area and cervical instability. The relevant indicators were observed on admission, at postoperative 1 week and 3 months, and at the last follow-up.

**Results:**

The operation time was 141.6 ± 13.7 min. The length of stay was 6.0 ± 2.5 days. VAS and JOA at different time points after operation were decreased compared with before operation (*p *< 0.05). There were no statistical differences between VAS or JOA at different postoperative time points (*p *> 0.05). The height, anteroposterior diameter and area of intervertebral foramen after operation were significantly increased compared with before operation (*p *< 0.05).

**Conclusion:**

Posterior cervical full-endoscopic technique shows the advantages of smaller invasion, faster recovery, significant effectiveness and fewer complications in our study. Meanwhile, it has little influence on the ROM and stability of the cervical spine. Therefore, it is a minimally invasive, safe and effective surgical method for the treatment of CSR with foraminal bony stenosis.

## Introduction

Cervical spondylotic radiculopathy (CSR) is mainly characterized by axial pain and/or radicular pain, which is caused by the compression of nerve roots due to cervical disc herniation or cervical spinal stenosis. The incidence rate of cervical spondylosis is about 3.8%–17.6%, of which CSR accounts for 60% of all types of cervical spondylosis (1). Nowadays, due to incentives such as using mobile phones and playing computer games excessively, CSR shows an increasing incidence in younger age. It has the characteristics of recurrent attacks, which seriously affects the life quality of patients and aggravates the economic burden. For most CSR patients, especially those with mild symptoms and short course of disease, conservative treatment is preferred (including life management, neck immobilization, physical therapy, drug therapy, etc.). However, some patients still have poor effectiveness and life quality after three months of conservative treatment. Surgical decompression is a better way for such people. In addition, early surgical treatment can also be considered for a few patients with serious conditions (2). Anterior cervical discectomy and fusion (ACDF) has been regarded as a classic operation for this disease, but the loss of cervical range of motion (ROM) and the subsequent degeneration and revision of adjacent segments after fusion often perplex spine surgeons (3). Posterior cervical foraminotomy (PCF) is another frequently-used method since it can preserve the motion segments while directly decompressing the focus. However, the posterior ligamentous complex injury and wound healing difficulties caused by extensive soft tissue dissection are also common thorny problems (4). In the past 30 years, spinal endoscopy has been developed vigorously and gradually applied to the treatment of cervical spondylosis. In 2005, Ahn et al. treated 17 cervical headache patients with total endoscopy through anterior approach and made a case series (5). Later in 2008, Ruttern et al. reported the success in treating CSR patients with endoscopic posterior transforaminal nucleus pulposus resection (6). Previous studies generally focus on CSR with soft disc herniation, while there are few studies on foraminal stenosis caused by ligament hypertrophy and osteophyte hyperplasia. In addition, if foraminal decompression is performed, it is necessary to expand the decompression range to ensure the sufficiency of decompression, and the subsequent problem of cervical spine stability has always been the hotspot of clinical discussion.

Therefore, our study aims to conduct a retrospective study using posterior cervical full-endoscopic technique for the treatment of CSR with foraminal bony stenosis, and evaluate its clinical effect and application value.

## Materials and methods

### Patient case selection and general information

We obtained ethical approval exemption from our ethics committee to perform this study since we didn't have direct contact with the participants. Patients treated for CSR in database records of our hospital were retrospectively analyzed from Dec 1, 2016, to Apr 30, 2020.

Inclusion criteria were as follows: (1) the diagnostic criteria referred to the CSR clinical guideline of NASS Evidence-Based Guideline Development Committee (7); (2) age from 18 to 75; (3) patients with single nerve root symptoms, and their cervical imaging data (including anteroposterior and lateral radiograph, hyperflexion and hyperextension radiograph, three-dimensional reconstruction CT and MRI) showed single-level and unilateral foraminal bony stenosis with bony herniation; (4) the nerve root symptoms were consistent with the focus location of the imaging data; (5) If necessary, angiography and block would be performed to identify the responsible nerve root; and (6) the conservative treatment failed to produce the desired results or the symptoms got progressively worse after 6 weeks, which seriously affected daily life and work. Exclusion criteria including: (1) patients with cervical segmental instability or nerve root disease; (2) patients with neck skin infection and history of posterior cervical surgery; (3) patients with central stenosis, spinal cord compression or symptoms of multi-level nerve root injury; (4) patients with severe systemic disease or organ dysfunction that cannot tolerate surgery.

### Surgical procedure and postoperative management

The posture refers to professor Ruetten's method (6). The patient's head is connected to the Mayfield head rest and fixed in hyperflexion position to open the posterior intervertebral space. Both lower extremities are in hip and knee flexion, and padded for protection.

**Figure 1:**
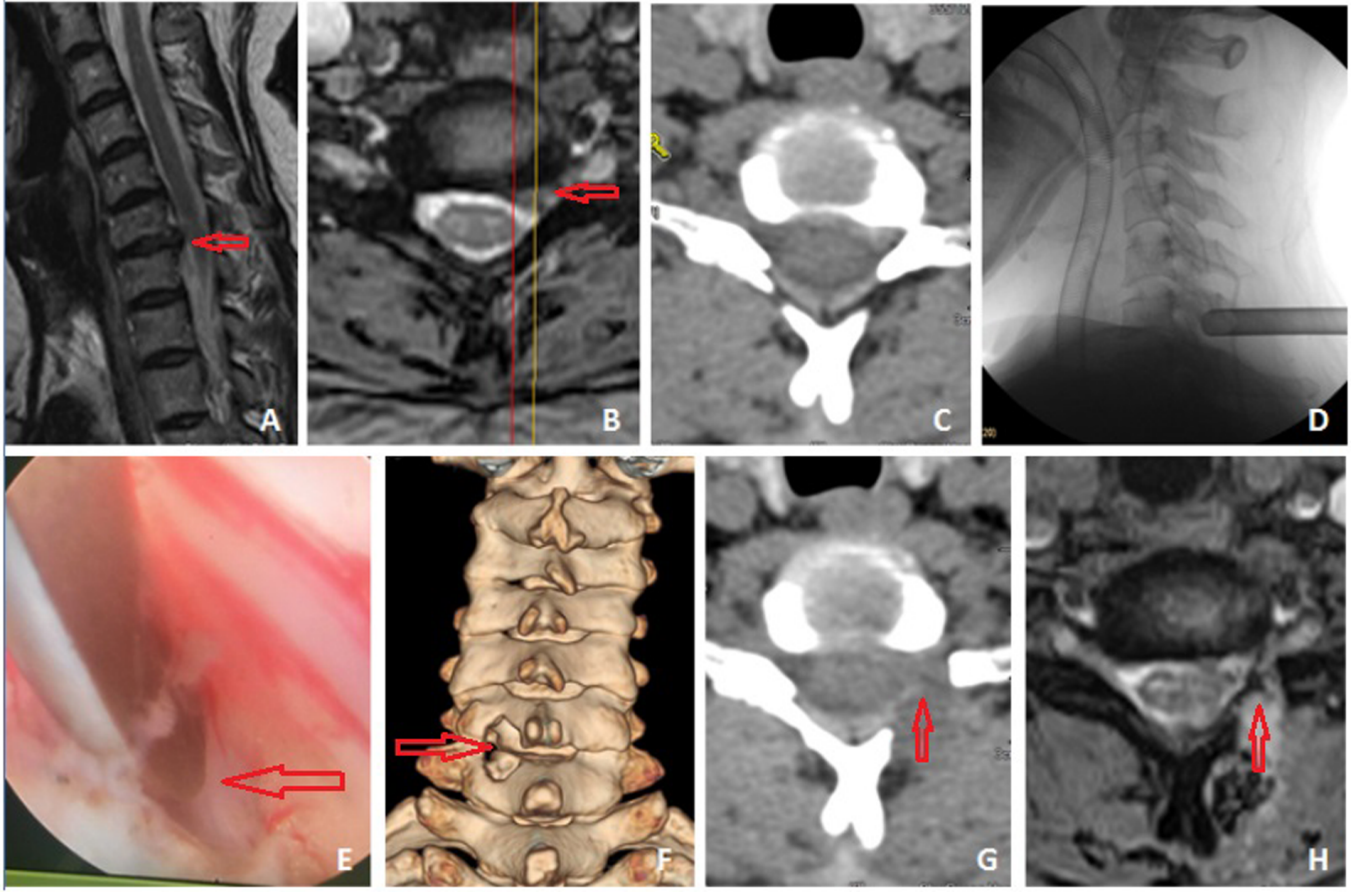
A patient with CSR (C6/7 left foraminal bony stenosis), female, 52 years old, was treated with posterior cervical full-endoscopic foraminotomy and decompression. Preoperative MRI and CT showed that C6/7 intervertebral disc protruded to the left rear, combined with left foraminal bony stenosis (**A–C**). After intraoperative localization, excising the herniated nucleus pulposus, cutting off the posterior osteophyte, performing foraminotomy and decompressing the nerve roots (**D,E**). Postoperative three-dimensional reconstruction CT showed a keyhole like decompression window in the left lamina of C6/7 (**F**) The postoperative axial CT and MRI showed satisfying decompression. The osteophytes and herniation had been removed, and the intervertebral foramen had been fully expanded (**G,H**).

The focus is located by anteroposterior and lateral radiograph. An 8 mm incision is made after local anesthesia. Place in and position the primary dilator accurately, then screw in the working sleeve. Subsequently, insert the endoscope. After connecting the lavage device, use nucleus pulposus forceps and a high-radiofrequency electrotome to clean the muscle and soft tissue on the surface of lamina. Once the “V” point is clearly identified, use a high-speed drill to expand the lamina space from head to tail. Generally, grind off the lower edge of upper lamina and the upper edge of lower lamina by about 3 mm respectively. Grind off the inner edge of lateral mass by 3 mm to the outside simultaneously. Remove the ligamentum flavum with basket forceps and gun forceps. Use the nerve hook to separate the scar on nerve and identify the spinal cord and exiting root. Next, grind off the upper edge of the lower cervical pedicle to create a potential channel from the axilla of nerve root to the rear of the intervertebral space. Gently rotate the working sleeve to protect the spinal cord and nerve root, adjust the lens to aim at the intervertebral space, excise the herniated nucleus pulposus and annulus fibrosus with nucleus pulposus forceps, and cut off the posterior osteophyte of the intervertebral space with an osteotome or a drill. Then explore again whether there is residual nucleus pulposus or osteophyte around the nerve root, perform annulus fibroplasty with the radiofrequency electrotome, and inject dexamethasone after hemostasis. Finally, remove the working sleeve followed by wound closure (Figure 1).

Routine swelling and pain relief and neurotrophic treatment are performed after the operation. Patients can begin out-of-bed activities with soft neck braces on the 2nd postoperative day. The neck brace should be worn for 4 weeks. The postoperative outpatient and telephone follow-up last for 6–18 months, with an average of 11.2 ± 6.5 months.

### Effectiveness evaluation

Operation time and length of stay were recorded. Relevant surgical complications such as infection, spinal cord and nerve injury, and systemic manifestations were also collected. The pain was evaluated based on visual analog scale (VAS). In addition, Japanese Orthopaedic Association (JOA) scores was used to assess the improvement in the motor, sensory and bladder dysfunction of patients. VAS and JOA score were recorded routinely on admission, at postoperative 1 week and 3 months, and at the last follow-up.

### Imaging evaluation

The intervertebral foramen height and anteroposterior diameter, and intervertebral foramen area were measured pre- and postoperatively (1 week). The sagittal section of the internal edge of the pedicle on operated side was defined as the measuring plane, and the height and diameter were measured based on this plane. For intervertebral foramen area, we transferring the thin-layer scanning DICOM data to the workstation (syngo.*via*, software version:VB40, Siemens Healthineers, Forchhiem, Germany), performing multiplanar reconstruction (MPR) in the vertical direction of the intervertebral foramen, manually found and determined the best display layer. Then we used the area measuring tool to sketch along the contour curve of the intervertebral foramen, and obtained the contour area (cm^2^) after drawing. Finally, we recorded the results.

In addition, cervical radiographs in hyperflexion and hyperextension position were collected before and 3 months after the operation. The evaluation standard of cervical instability (angular displacement larger than 10° or horizontal displacement larger than 3.0 mm) was used to judge whether there was postoperative segmental instability (8).

### Statistical analysis

Statistical Packages of Social Sciences (SPSS) software (version 22.0) was used to analyze the collected data. Measurement data were tested for normality of distribution. Data with normal distribution were expressed as mean ± standard deviation (`x ± s). Counting data were described as percentages or constituent ratios, and were analyzed using the *χ*^2^ test, Fisher's exact probability method, or non-parametric testing. Differences in variables before and after the operation were analyzed through one-way ANOVA or paired *t*-test. Meanwhile, *p *< 0.05 was considered statistically significant.

## Result

A total of 22 consecutive cases from our database of patients were involved in the current study, including 15 males and 7 females, with an average age of 49.6 ± 9.2 years (ranged from 36 to 78 years). All cases were single-level unilateral CSR with foraminal bony stenosis, including 1 case of C3/4 level, 4 cases of C4/5 level, 15 cases of C5/6 level and 2 cases of C6/7 level (Table 1).

**Table 1:** General information of the patients.

No.	Clinical Information	Number
1	**Sex (Male/Female)**	15/7
2	**Age (Years)**	49.6 ± 9.2
3	**Affected Level**	
	C3/4	1
	C4/5	4
	C5/6	15
	C6/7	2
4	**Operation Time (Min)**	141.6 ± 13.7
5	**Length of Stay (Day)**	6.0 ± 2.5
6	**Wound Healing (I Intention/Others)**	22/0
7	**Surgical Complications**	
	Infection	0
	Hematoma	1
	Nerve or Spinal Cord Injury	0

### Perioperative conditions

The radiating pain and neurological symptoms of all cases were significantly improved after operation. There were no serious complications such as spinal cord and nerve injury. The operation time was 141.6 ± 13.7 min and the length of stay was 6.0 ± 2.5 days. All wounds healed by first intention without postoperative infection. One case suffered a transient burning sensation of the affected extremity after operation, and the pain was not significantly relieved compared with that before operation. CT and MRI showed that the decompression was sufficient, but there was a low signal area in the intervertebral foramen on MRI. The patient was given fluid gelatin to stop bleeding during operation, and it was considered that the excessive fluid gelatin and insufficient lavage resulted in local hematoma. Intravenous dexamethasone, dehydration and analgesic treatment were given postoperatively. The patient's pain was significantly improved on the 3rd day after operation, and all pathological symptoms had disappeared at the postoperative 3-month follow-up (Table 1).

### Effectiveness analysis

VAS scores and JOA at different time points after operation were decreased compared with before operation (*p *< 0.05), which indicated significant alleviation of pain and improvement of motor function. There were no statistical differences between VAS scores or JOA at different postoperative time points (*p *> 0.05) (Table 2).

**Table 2:** Comparison of VAS and JOA before and after operation.

Index	Preoperative	Postoperative 1 week	Postoperative 3 months	Last follow-up
VAS	8.09 ± 1.24	2.14 ± 1.83^a,b^	1.68 ± 0.92^a,b^	1.23 ± 1.05^a,b^
JOA	11.91 ± 6.37	15.18 ± 4.62^a,b^	15.82 ± 5.38^a,b^	16.27 ± 3.94^a,b^

^a^ *p *< 0.05 vs. preoperative.

^b^ *p *> 0.05 vs. different postoperative time point.

### Imaging analysis

Three-dimensional CT showed that the postoperative intervertebral foramen height and anteroposterior diameter were increased compared with before operation (*p *< 0.05). The postoperative intervertebral foramen area was significantly larger than that before operation (*p *< 0.05), which clearly indicated that the spinal canal and intervertebral foramen are fully decompressed under endoscope (Table 3).

**Table 3:** Comparison of intervertebral foramen parameters before and after operation.

Index	Preoperative	Postoperative 1 week
**Intervertebral foramen**		
Height	9.08 ± 3.45	12.39 ± 5.81^a^
Anteroposterior diameter	4.19 ± 2.37	9.05 ± 3.62^a^
Area	28.71 ± 10.65	126.64 ± 27.58^a^

^a^ *p *< 0.05 vs. preoperative.

In addition, according to the criteria provided by the literature (8), all cases had no signs of segmental instability at the postoperative 3 months' follow-up.

## Discussion

PCF is an ideal method that can preserve the motion segment and decompress the focus simultaneously. The early PCF usually requires extensive dissection and exposure which result in damage to the muscles, ligaments and soft tissues of the posterior cervical spine. Therefore, postoperative axial pain and cervical instability are common complications (9, 10). The development of channel and microscopic technique promotes the minimally invasive incision and visual operation of PCF. On the premise of reducing soft tissue dissection, more sophisticated action is carried out to ensure the efficacy and safety of the operation. However, the placement angle of the channel technique is limited by the occlusion of the spinous process, and the microscope has the defect of blind area, thus it is often difficult to treat complicated cases of intervertebral foraminal stenosis (11–13). Compared with the previous methods, the cervical endoscopic technique is more minimally invasive and has a wider field of vision under the endoscope. Moreover, it can obtain a greater angle of view with the “joystick” technique (14). Since endoscopic PCF was carried out by professor Ruetten for the first time in 2008, its safety and effectiveness have been repeatedly verified, and it has become the first choice for non-fusion surgery in the treatment of CSR.

The pathological basis of CSR is the compression of cervical nerve root caused by the intervertebral foraminal stenosis, resulting in a series of nerve dysfunction. Clinically, the foraminal stenosis is often caused by the herniation and calcification of intervertebral disc, the hyperplasia and ossification of Luschka joint, and the hypertrophy and adhesion of ligamentum flavum. In the treatment of CSR, ACDF usually indirectly decompresses the foramen by prop open the intervertebral space. Mostly, for complicated cases of foraminal stenosis, it is necessary to partially or completely remove the Luschka joint in order to decompress the cervical foramen to achieve the therapeutic effect. However, the removal of the Luschka joint may lead to the prosthesis subsidence and the cervical instability. Therefore, whether the Luschka joint should be resected is still controversial in the clinical practice (15, 16). PCF can not only remove the herniated intervertebral disc, but also grind off the posterior osteophyte and loosen the adhesion around the nerve root under the premise of preserving the Luschka joint. With the advantage of endoscope, PCF can preserve the stability of posterior structure as much as possible and achieve the full decompression of nerve root. The results of this study show that after endoscopic foraminal decompression, the intervertebral foramen height, the anteroposterior diameter and the intervertebral foramen area are all increased compared with those before operation, indicating that endoscopic PCF can fully decompress the nerve and intervertebral foramen. Meanwhile, the postoperative VAS and JOA score of the patients are also improved. Their pain and neurological symptoms are significantly relieved without serious complications and sequelae.

Sufficient decompression of cervical spinal canal and intervertebral foramen through posterior approach is an important basis to ensure the clinical effect. However, if more than 1/2 of the facet is resected, the postoperative cervical spine may be unstable. Raynor's research on cadaver specimens shows that if 70% of bilateral cervical facet joints is resected, fracture would occur under the compression of 159 lb, while fracture will not occur under the compression of 208 lb when 50% of bilateral facet joints is resected (17). Therefore, how to achieve sufficient decompression under the premise of preserving the facet and pedicle as much as possible is the key to the surgical efficacy. Due to the limitation of visual angle, when using microscope and minimally invasive channel, the surface of facet must be resected to perform the decompression of foramen and medial edge of pedicle. By comparison, the lens of the endoscope is located at the head end. Therefore, combined with the “joystick” technique, the thin body of the endoscope can make it easier to deeply decompress the medial edge of facet joints and the upper and medial edge of the pedicles without sacrificing too much bone of facet joints. Moreover, if the anterior herniation is wrapped or ossified, the high-speed drill or endoscopic osteotome can also be used to remove the intervertebral disc under the condition that protecting the nerve root with endoscopic sheath. The current study shows no cervical instability at the corresponding segment in all cases at the last follow-up, which proves that the posterior cervical full-endoscopic technique can fully guaranteed the stability of the operative segment.

Through the summary of the cases, we believe that the posterior cervical full-endoscopic technique has the following advantages: (1) smaller invasion, less injury to the facet joints and posterior soft tissue, and better preservation of the stability of the cervical spine; (2) the shorter length of stay and the earlier functional training, which can not only enhance recovery after surgery, but also save medical resources; (3) preserving the motion segment, which greatly reduces the influence on ROM of the cervical spine and the degeneration of adjacent segments; (4) The magnification effect of endoscopic visual field and continuous hydraulic pressure can effectively maintain the clear view of surgery, in order to increase the identification of tissue structure and reduce the risk of nerve and vascular injury; (5) the 360° intervertebral foraminotomy can achieve fully decompression under minor interference of the nerve root.

Admittedly, there are some deficiencies in the posterior cervical full-endoscopic technique, especially the long learning curve. The technique places enormous demands on operation under endoscope and use of grinding drill. Those with insufficient experience performing the surgery blindly will cause common complications including incomplete decompression, nerve root irritation, infection, etc., or even disastrous consequences such as spinal cord injury and quadriplegia (18, 19). In this study, 1 case (4.5%) had postoperative burning sensation and serious pain of the affected extremity. After active dehydration, antiphlogistic and analgesic treatment, the symptoms gradually improved. We analyzed that it was the local hematoma compressing the nerve root which led to the complication.

The current study has several limitations and it could be altered in some ways to better ascertain the conclusion. First of all is its relatively small sample size. We limited the indications to foraminal bony stenosis in order to treat CSR more accurately. However, appropriate extension of indications and larger sample population will allow for more meaningful statistical testing and smaller deviation. Another limitation is the short follow-up period. The longest period in this study was 18 months since we initially believed that longer period of follow-up would reduce patients' compliance and make data collection more difficult. Nevertheless, longer period up to 2 or 3 years may improve the reliability of evidence and be more likely to find the occurrence of adjacent segment disease, so as to confirm the long-term effect of this method. Therefore, large sample, multicenter and long follow-up studies should be performed in our future clinical work to evaluate its clinical application value.

## Conclusion

Posterior cervical full-endoscopic technique shows the advantages of smaller invasion, faster recovery, significant effectiveness and fewer complications in our study. Meanwhile, it has little influence on the ROM and stability of the cervical spine. Therefore, it is a minimally invasive, safe and effective surgical method for the treatment of CSR with foraminal bony stenosis. However, more improved studies should be conducted to provide orthopedic surgeons with the best evidence-based information.

## Data Availability

The original contributions presented in the study are included in the article/Supplementary Material, further inquiries can be directed to the corresponding author/s.
